# Electrical signalling on Bt and non-Bt cotton plants under stress by *Aphis gossypii*

**DOI:** 10.1371/journal.pone.0249699

**Published:** 2021-04-08

**Authors:** Jéssica K. S. Pachú, Francynes C. O. Macedo, José B. Malaquias, Francisco S. Ramalho, Ricardo F. Oliveira, Flávia Pereira Franco, Wesley A. C. Godoy

**Affiliations:** 1 Department of Entomology and Acarology, Luiz de Queiroz College of Agriculture (ESALQ), University of São Paulo (USP), Piracicaba, São Paulo, Brazil; 2 Department of Biological Sciences, Luiz de Queiroz College of Agriculture (ESALQ), University of São Paulo (USP), Piracicaba, São Paulo, Brazil; 3 Department of Biostatistics, São Paulo State University (UNESP), Botucatu, Brazil; 4 Biological Control Unit ⁄ Embrapa Algodão, Campina Grande, Paraíba, Brazil; Zhejiang University, CHINA

## Abstract

Plants have developed various mechanisms to respond specifically to each biotrophic attack. It has been shown that the electrical signals emitted by plants are associated with herbivory stress responses and can lead to the activation of multiple defences. Bt cotton is a genetically modified pest-resistant plant that produces an insecticide from *Bacillus thuringiensis* (Bt) to control Lepidopteran species. Surprisingly, there is no study–yet, that characterizes the signalling mechanisms in transgenic cotton plants attacked by non-target insects, such as aphids. In this study, we characterized the production of electrical signals on Bt and non-Bt cotton plants infested with *Aphis gossypii* and, in addition, we characterized the dispersal behaviour of aphids to correlate this behaviour to plant signalling responses. Electrical signalling of the plants was recorded with an extracellular measurement technique. Impressively, our results showed that both Bt and non-Bt cotton varieties, when attacked by *A*. *gossypii*, emitted potential variation-type electrical signals and clearly showed the presence of distinct responses regarding their perception and the behaviour of aphids, with evidence of delay, in terms of signal amount, and almost twice the amount of Cry1F protein was observed on Bt cotton plants at the highest density of insects/plant. We present in our article some hypotheses that are based on plant physiology and insect behaviour to explain the responses found on Bt cotton plants under aphid stress.

## Introduction

An organism’s capacity to survive in an ecosystem depends on its ability to respond quickly and efficiently to external stimuli and to develop effective and sustainable defences [1]. For this reason, plants have developed numerous mechanisms to react specifically to each biotrophic attack, and cell-to-cell communication between distant tissues is essential to coordinate activities in response to the environment. Thus, plants need to produce a signalling mechanism to integrate perception, transmission, and response to biotic and abiotic actions that occur in the ecosystem [2–5]. Electrical signals have been shown to be associated with responses to herbivory [6], leading to the activation of multiple organism defences [7]. However, studies that better characterize the electrical signalling mechanisms in plants attacked by herbivores, such as aphids, are still scarce.

The aphid *Aphis gossypii* Glover (Hemiptera: Aphididae) is a cotton-damaging pest [8] of cotton. and one of the most important non-target species of Bt cotton, which is a genetically modified variety expressing proteins derived from *Bacillus thuringiensis* (Berliner) (Bt), which gives them high efficiency against some lepidopteran species [9, 10]. However, researchers have raised concerns about their potential impact on nontarget organisms such as aphids [11], and their stress on the Bt and non-Bt plant physiology [12].

Different environmental stimuli cause specific responses in living cells that are capable of transmitting electrical signals [13, 14]. Among the signals involved in electrophysiological responses, variation potentials are characterized by rapid depolarization and subsequent slow repolarization. The amplitude and shape of the variation potentials (VPs) vary with the stimulus intensity. In addition, the magnitude and speed of responses decrease as the signal moves away from the stimulus site, and its induction depends on the type of damage sustained [15].

Although the methods used to produce transgenic crops are being continuously improved, the relationship of plant-insect can be influenced. Thus, it is crucial to understand how plants produce different signs of stress and convert them into appropriate specific responses [12]. Therefore, it is important to characterize the type of electrical signalling of Bt and non-Bt cotton plants as a function of the stress caused by *A*. *gossypii* and provide insights to understand how plants convert these different signals into appropriate physiological reactions. In our study, we characterized the production of electrical signals on Bt [variety WideStrike®] cotton plants and their non-Bt isoline [variety FM 993] infested with *A*. *gossypii* in alternating light–dark cycles. The aphid *A*. *gossypii* was used as a model insect for the study because insect feeding occurs at the phloem level, and the biological interactions between the herbivore and its host plant can be considered unique. Additionally, we characterized *A*. *gossypii* dispersal behaviour to relate it to plant signalling responses.

## Methods

### Characterization of the electrical signalling potential of Bt and non-Bt cotton plants

*Aphis gossypii* were reared at the Insect Ecology and Forestry Entomology Laboratory of the Department of Entomology and Acarology (LEA) of the Luiz de Queiroz College of Agriculture (ESALQ) at the University of São Paulo Paulo (USP), Piracicaba, São Paulo, Brazil. Adults of *A*. *gossypii* were collected in cotton plants in the experimental area from LEA. Specimens of *A*. *gossypii* were transported to the laboratory for the establishment of rearing at LEA. Insect-rearing stocks were kept by 2 generations in a phytotron chamber at 26±1°C, with relative humidity of 60±10% and photophase of 12 h.

Cotton plants expressing Cry1Ac/Cry1F [variety FM 975 (WideStrike®)] and their non-Bt isoline [variety FM 993] were used in this study. The cotton plants were planted in plastic pots (one plant per pot) 25 cm in diameter and 40 cm in height, containing soil conditioning substrate (Forth®) and kept in a phytotron chamber at 26±1°C, with relative humidity of 60±10% and photophase of 12 h.

Bioassays to record electrical signalling of cotton plants were conducted in the laboratory. The experimental design was a randomized block design, and each treatment was repeated 10 times = 10 plants per treatment. The measurement of electrical signals was made on the Bt or non-Bt cotton plant surface that reached the six-leaf stage. A technique was used to detect electrical signalling potential differences over long periods. At the time of the electrical signal measurements, the cotton plants were placed in a Faraday cage to ensure electromagnetic isolation of the environment at 26±1°C, with a relative humidity of 60±10% and a 12-h photophase.

**Fig 1:**
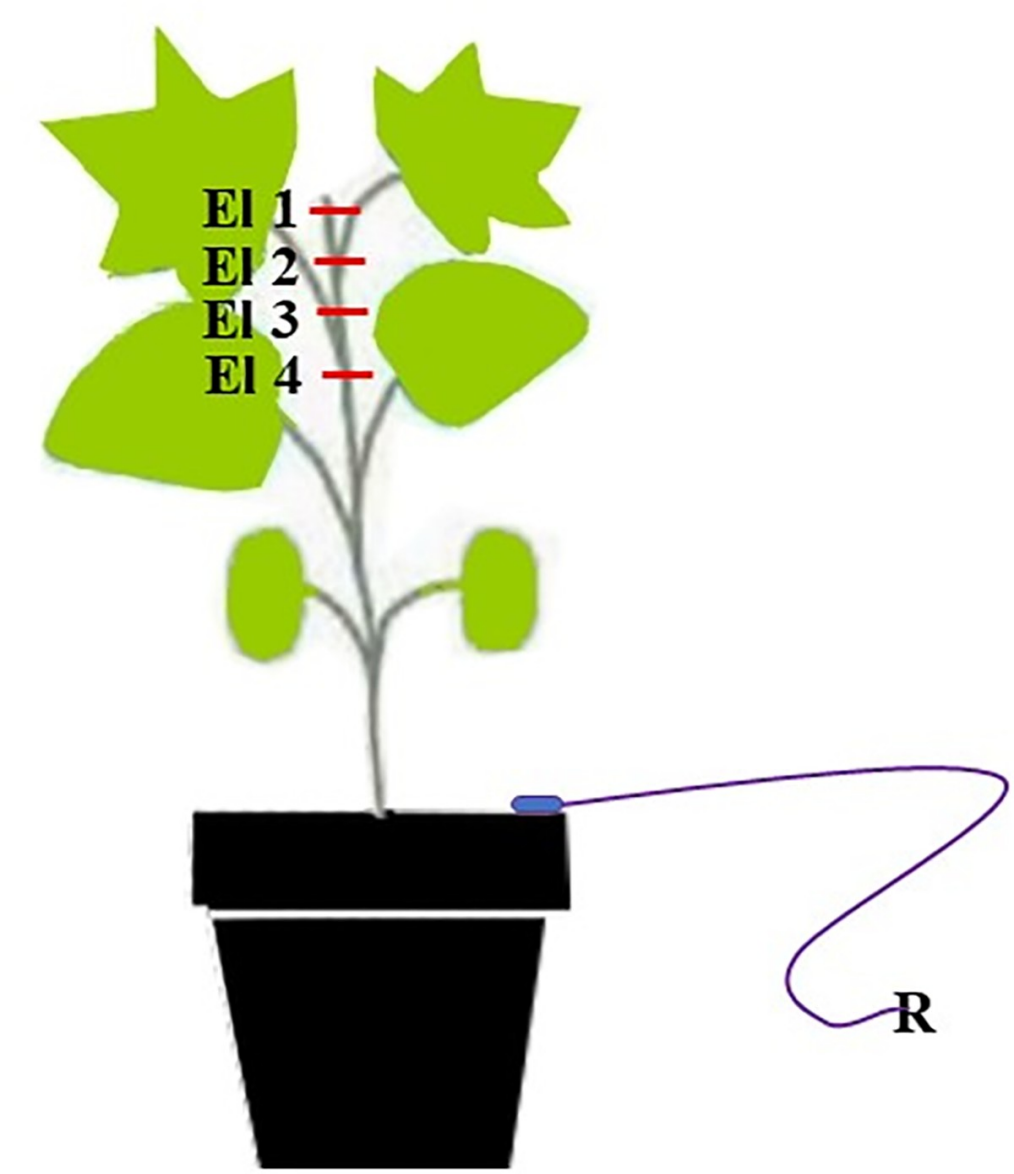
Scheme of the electrodes (El, E2, E3 and E4) in the cotton plant inserted into the stem; and R (reference electrode) inserted into the soil.

Measurements were made using electrodes consisting of a 0.25–0.5 mm diameter silver lead wire chlorinated in 3 M KCl solution. After the acclimatization period, five electrodes were used. Four of which were inserted in different arrangements along the stem of cotton plants. The reference electrode (fifth electrode), that has the same composition of the recording electrodes, was inserted in the ground (Fig 1). The electrodes are connected to a four-channel data acquisition system with a built-in amplifier (World Precision Instruments Lab-Trax-4 / 24T model) that is connected to a computer with LabScribe^®^ version 3.0 software that decodes the signal [13, 16–18].

The recordings of electrical activities in cotton plants started one hour after the electrode insertion. The records were performed continuously for three days. The following variables were obtained: amplitude of the signal, number of signals generated, time after insect infestation to emission of the signals and frequency of signals generated by cotton plants. The electrical signalling profile was contrasted between Bt and non-Bt cotton plants infested with those not infested with *A*. *gossypii* (control).

Bt and non-Bt cotton varieties were planted in plastic pots containing soil conditioning substrate (Forth^®^) and kept separately in cages under the same climate conditions mentioned before. The plants were infested with the aphids with a paintbrush. We used the following 6 treatments: **a1**. Bt cotton plants infested with 30 aphids/plant; **a2**. Bt cotton plants infested with 60 aphids/plant; **a3**. Bt cotton without aphid (Bt cotton control); **a4**. non-Bt cotton plants infested with 30 aphids/plant; **a5**. non-Bt cotton plants infested with 60 aphids/plant; and **a6**. non-Bt cotton without aphid (Bt cotton control).

### Dispersal pattern of *A*. *gossypii* in Bt and non-Bt cotton plants

Bioassays were performed to study aphid behaviour and associate it with data obtained from electrical signalling bioassays. A randomized block design, distributed in 10 blocks = 10 plants/treatment, with four treatments was used: **a1**. Bt cotton plants infested with 30 aphids/plant; **a2**. Bt cotton plants infested with 60 aphids/plant; **a3**. non-Bt cotton plants infested with 30 aphids/plant and **a4**. non-Bt cotton plants infested with 60 aphids/plant. Bt and non-Bt cotton varieties were planted in plastic pots containing soil conditioning substrate (Forth^®^) and kept in the same climate conditions mentioned before.

Aphid infestations were performed on Bt and non-Bt cotton that reached the six-leaf stage. We used a paintbrush to infestation of the plants with the aphids. The plants were divided into the following equidistant three regions: bottom, middle and top. The number of aphids was recorded in each plant region at 0 (immediately during infestation), 24, 48 and 72 h after infestation. To evaluate aphid dispersal behaviour as a function of varieties (Bt and non-Bt cotton) and aphid densities, the negative binomial distribution parameter *k* was used.

There are three basic spatial pattern distributions: random distribution, regular or uniform distribution, and aggregate or contagious distribution. This parameter *k* is an indicator of uniform distribution, where when *k* tends to zero, the distribution is highly aggregated, *k* ranging from 2 to 8 indicates moderate aggregation, and values greater than 8 (*k* > 8) indicate that the distribution is random (39). The *k* values were estimated by the method of moments (a statistical method for constructing an estimator).

### Data analyses

#### Characterization of electrical signalling potential of Bt and non-Bt cotton plants

Descriptive analyses were conducted with boxplots aiming to characterize the quantiles, medians, maximum and minimum values, and outliers of the variables and time for the emission of VPs (variation potentials) after the infestations with aphids on Bt and non-Bt cotton plants and amplitude of VPs.

Correlation analyses were conducted between the variables VP amplitude and signal emission time after infestation plants within each cotton variety. The degree of correlation between the variables in each condition was studied using Spearman’s rank correlation coefficient (P <0.05) using the cor.test function of the R program.

Data on the number of signals per time interval after infestation of Bt and non-Bt cotton plants with aphids were subjected to deviance analysis, with the purpose of studying the interaction involving cotton variety, aphid / plant density and time interval. A generalized linear model with a quasi-Poisson distribution was used. The goodness of fit of the model was evaluated with a simulated normal envelope using the hnp package in the R program [17].

Deviance analysis was applied to study the interaction involving cotton variety, aphid/plant density and period (photophase / scotophase) in the number of VPs. Data were divided into four sections, three of which corresponded to the data recorded during three days of observation, and the last section corresponded to the accumulated data recorded during the three days of evaluation. Negative binomial generalized linear models were used for approximately the 1st and 2nd evaluation days, while quasi-Poisson models were adopted for data recorded on the 3rd day and total accumulated over the three days of evaluations. We used a half-normal plot with a simulated envelope with the hnp package [17] to assess the goodness-of-fit of the models.

### *Aphis gossypii* dispersal pattern on Bt and non-Bt cotton plants

The parameter *k* in each cotton variety and density was compared by confidence intervals. Confidence intervals were generated from the values of *k* for each block. We used the nonparametric bootstrap technique, with 10,000 pseudoreplications, and for the resampled parameter in each treatment, we used the R program boot package [18].

#### Multinomial analysis

The probability of aphids occurring within each region of Bt and non-Bt cotton plants in each treatment (variety and aphid density) was estimated and compared with a multinomial linear model. The analyses to estimate the probabilities and their comparisons were conducted with nnet [19] and emmeans [20] packages from R.

## Results

### Characterization of the electrical signalling potential of Bt and non-Bt cotton plants

**Fig 2:**
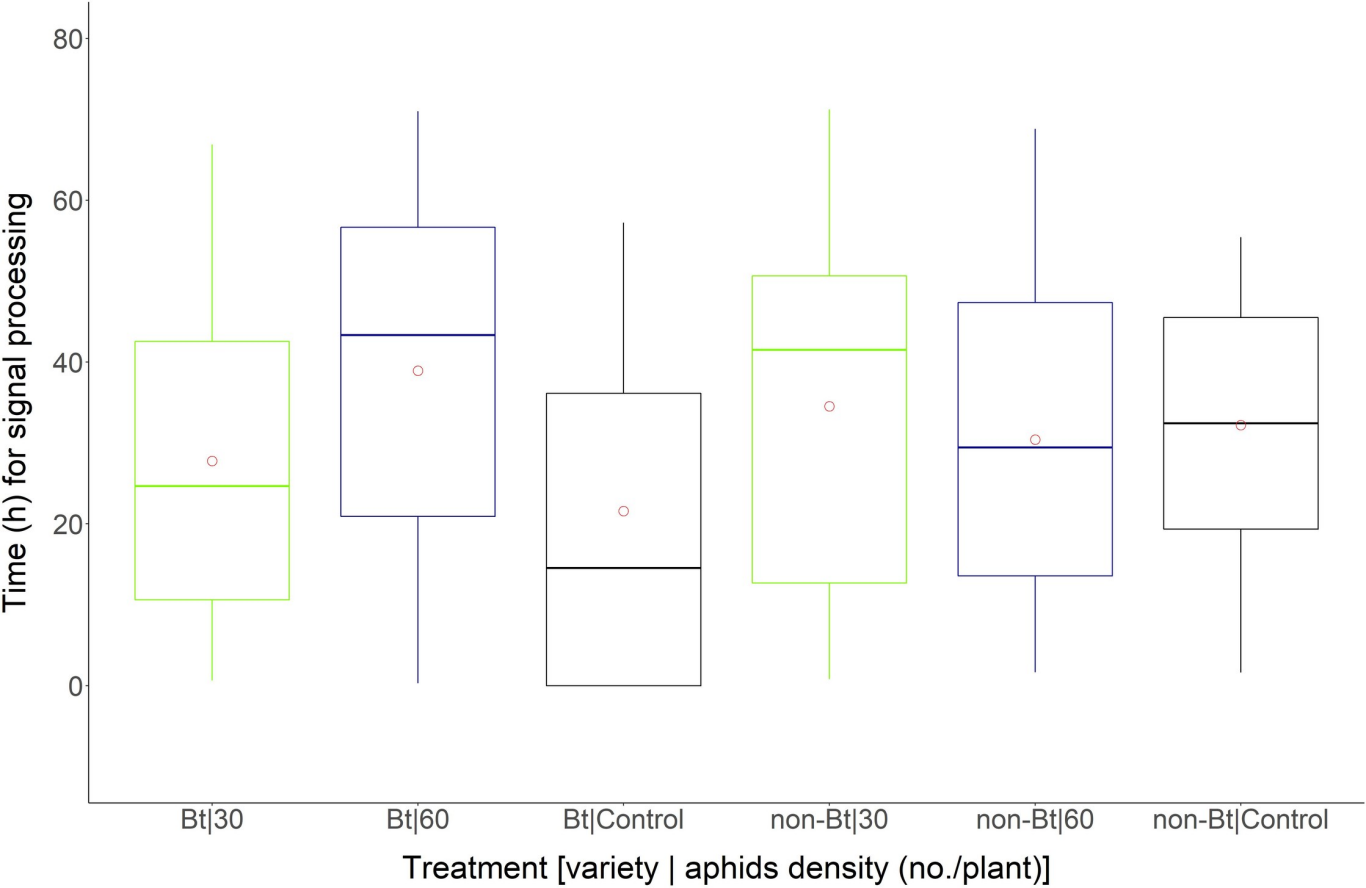
Boxplot of time (h) of exposure of Bt and non-Bt cotton plants to different densities of *A*. *gossypii* emitting potentials of variation (VPs) (mV). Bt and non-Bt cotton plants were infested at densities of 30 and 60 aphids/plant and in the absence of aphids (Control). Circle within the boxplot corresponds to mean of time for each treatment.

In descriptive analysis, it was possible to visualize that Bt cotton plants when infested with *A*. *gossypii* emitted the first VPs (“minimum value” in boxplot) between time intervals of 0.31 h (60 aphids/plant) and 0.64 h (30 aphids/plant) (Fig 1). In the absence of aphids, only two Bt cotton plants emitted these electrophysiological signals. Non-Bt cotton plants emitted the first VPs (“minimum value” in boxplot) after 0.80 h when kept at 30 aphids/plant and after 1.60 h at the density of 60 aphids/plant (Fig 2).

The Bt and non-Bt cotton plants infested with aphids emitted signals after 60 h of aphid infestation (Fig 2), while in cotton plants used as a control, the maximum signal emission values were observed at 55 and 57 h in non-Bt and Bt cotton plants, respectively (Fig 2).

**Fig 3:**
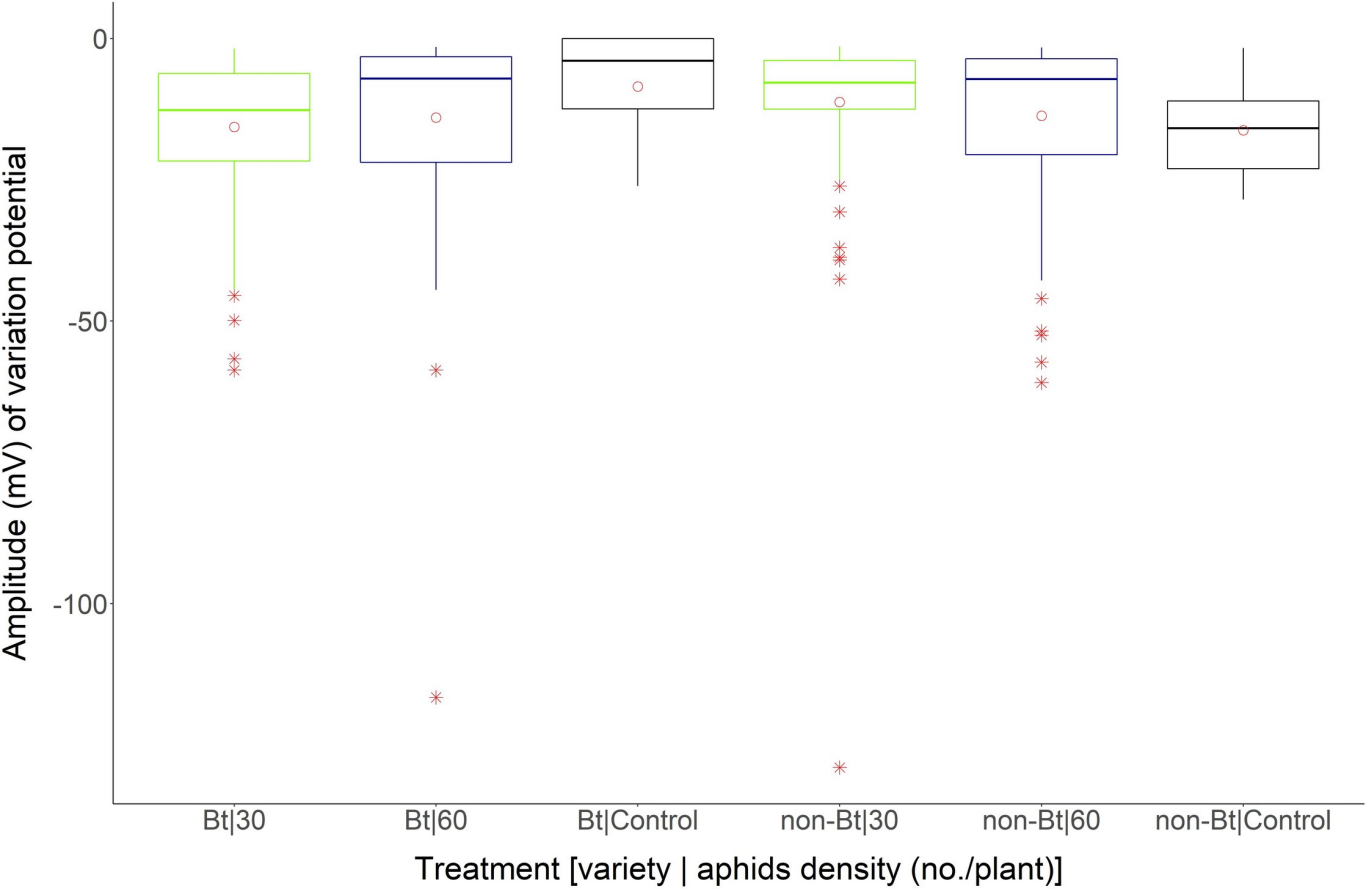
Boxplot of amplitude (mV) of variation potentials in Bt and non-Bt cotton plants exposed to different densities of *A*. *gossypii* Bt and non-Bt cotton plants exposed to densities of 30 and 60 aphids/plant and in the absence of aphids (Control). Circle within the boxplot corresponds to mean of time for each treatment. Asterisk out of the boxplot corresponds to outlier for each treatment.

The maximum amplitude (mV) (“maximum value” in boxplot) of VP found in the control cotton plants was near -28 mV. In general, the mean amplitude (mV) of VP (points within the boxplots) was near all treatments, ranging from -17 mV (Bt–control cotton plants) to -11.22 mV (non-Bt cotton plants infested with 30 aphids/plant). Outliers (points out of boxplots) occurred for 30 aphid/plant (-129 mV) infested non-Bt cotton plants and 60 aphid/plant (-116.60 mV) infested Bt cotton plants (Fig 3).

Spearman rank analysis revealed that there was no correlation between the amplitude (mV) of VP of VP and time (h) to emission of signals by cotton plants after aphid infestation at all densities studied within each variety (Bt and not Bt), except at the densities of 30 aphids / non-Bt cotton plants (*ρ* = -0.2659; *P* = 0.0060) and 60 aphids/Bt cotton plants (*ρ* = - 0.3528; *P* = 0.00254).

Analyzing the number of VPs emitted by the cotton plants, we observed that infestation-free plants emitted few signals, with an average accumulation of 0.75 (control–Bt cotton) and 2.50 signals (control–non-Bt cotton) over 72 h. Only two Bt cotton plants emitted electrical signals in the absence of aphid stress (Table 1).

**Table 1:** Number of variation potentials emitted (mean ± SE) by Bt and non-Bt cotton plants when subjected to different exposure times and densities of *A*. *gossypii*/plant.

Time interval (h)	Bt cotton			non- Bt cotton		
	0 (control)	30	60	0 (control)	30	60
0‒12	0.00 ± 0.00^*nia*^	6.0 ± 2.17 A a	3.50 ± .89 A b	0.50 ± 0.50^*nia*^	6.00 ± 2.08 A a	6.25 ± 2.39 A a
12‒24	0.00 ± 0.00^*nia*^	4.50 ± 1.04 B a	1.75 ± 0.47 C b	0.00 ± 0.00^*nia*^	4.75 ± 1.75 AB a	4.50 ± 1.25 BC a
24‒36	0.50 ± 0.25^*nia*^	2.75 ± 0.62 C b	1.00 ± 1.00 D d	0.75 ± 0.47^*nia*^	1.75 ± 0.75 C c	5.25 ± 1.88 AB a
36‒48	0.00 ± 0.00^*nia*^	4.75 ± 1.25 B a	3.50 ± 1.04 A b	0.50 ± 0.28^*nia*^	3.75 ± 0.94 B ab	3.50 ± 0.86 C b
48‒60	0.25 ± 0.25^*nia*^	1.25 ± 0.62 D c	2.50 ± 0.50 B b	0.75 ± 0.75^*nia*^	5.75 ± 0.85 A a	5.00 ± 1.00 AB a
60‒72	0.00 ± 0.00^*nia*^	2.25 ± 1.65 C b	4.00 ± 0.08 A a	0.00 ± 0.00^*nia*^	4.25 ± 1.54 AB a	1.50 ± 0.64 D c
*Ʃ*_*accumulated*_ (total)	0.75 ± 0.25^*nia*^	22.00 ± 0.17 a	16.25 ± 0.13 b	2.50 ± 0.50^*nia*^	26.25 ± 0.21 a	26.00 ± 0.20 a

Capital letters compare averages within each column, and lowercase letters compare averages within each row. Means followed by the same letters do not differ from each other by overlapping confidence intervals generated by the quasi-Poisson model (*P* <0.05). ^*nia*^ = not incorporated in the analysis, because the absence of variability.

In the accumulated emission of VPs over 72 h, it was verified that Bt cotton plants exposed to 60 aphids/plant density emitted fewer signals compared to the other conditions (*P* < 0.05) under aphid stress. However, by assessing the emission within the intervals, the deviance analysis revealed that the signal emission pattern in each variety was influenced by the time interval and aphid density, as there was a significant interaction between these three factors (*P* = 0.0488) (Table 1).

**Fig 4:**
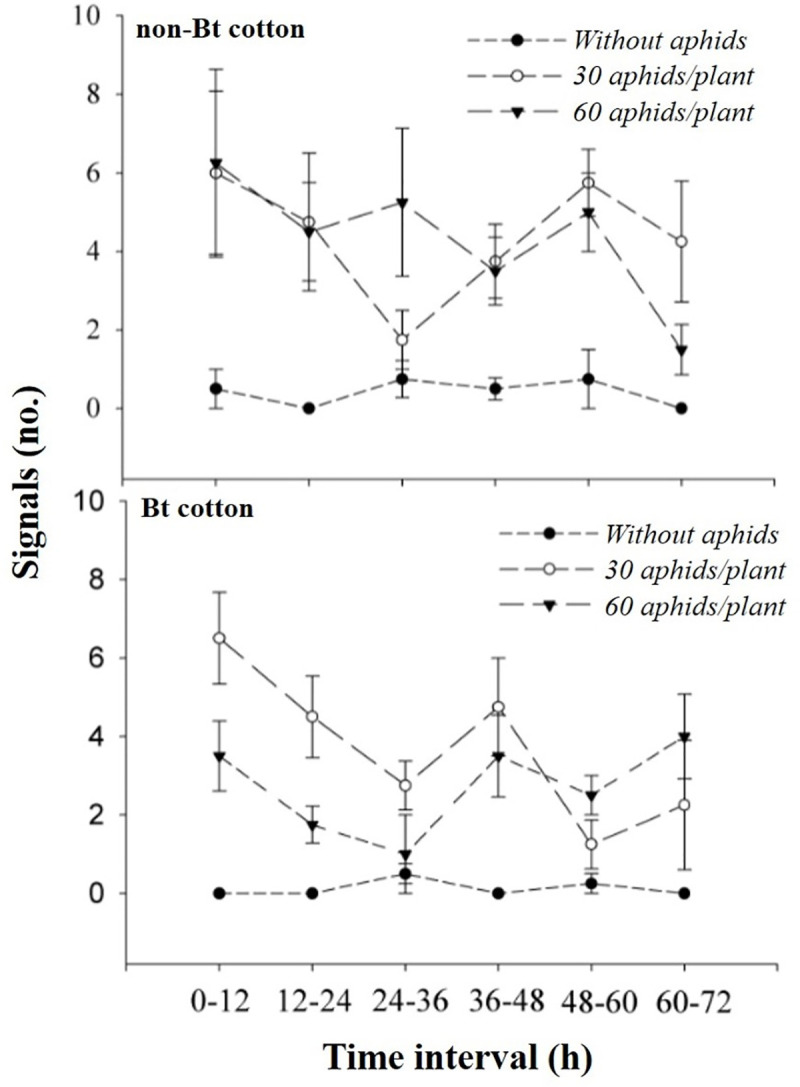
Variation potentials emitted (no.) (MEAN ± SE) by Bt and non-Bt cotton plants at different time intervals (h) after infestation of 30 and 60 aphids / plant and control (absence of aphid). Mean data (points) and error bars (SE) predicted by the generalized linear quasi-Poisson model, except for the plotted values for the control used in both cotton varieties (Bt or not Bt).

The highest number of signals emitted by Bt cotton plants when exposed to aphids occurred in the time interval after infestation of 0–12 h (30 aphids/plant) and 0–12, 36–48 and 60–72 h (60 aphids/plant) (Table 1, Fig 4).

When we compared the signal emission pattern between combined treatments involving aphid densities and cotton varieties within each time interval, it was possible to verify a delay in terms of the production pattern of signalling on Bt cotton plants under stress with 60 insects/plant because until the time interval of 36 h after infestation, there was a lower signal emission by Bt cotton plants when exposed to 60 aphids/plant in relation to the other conditions of aphid density/Bt or non-Bt cotton variety (Table 1, Fig 4).

In the time interval of 36‒48 h, the emission of signals by Bt cotton plants was lower only in relation to Bt cotton with 30 aphids/plant. Additionally, in the time interval of 60‒72 h, the signal production by cotton plants was higher when the Bt and non-Bt cotton plants were exposed to densities of 60 and 30 aphids, respectively, in relation to other conditions (Table 1, Fig 4).

The deviance analysis on the interaction of the factors: aphid density *versus* cotton variety *versus* light period within each studied day (1st, 2nd or 3rd day) and accumulated over these three days influencing the number of VPs emitted by plants shows that there was no interaction (P> 0.05) among the studied factors for the 1st and 2nd day and the accumulated days of exposure of Bt and non-Bt cotton plants to aphids. The factor density [*F*_density_ = 2.29, *P*_density_ = 0.1294 (1st day); *F*_density_ = 0.0070, *P*_density_ = 0.95 (2nd day); *F*_density_ = 0.1734, *P*_density_ = 0.6813 (cumulative total)], variety [*F*_variety_ = 0.003, *P*_cultivate_ = 0.95 (1st day); *F*_variety_ = 3.0896, *P*_variety_ = 0.07 (2nd day); *F*_variety_ = 2.2726, *P*_variety_ = 0.1466 (cumulative total)] and period [*F*_period_ = 1.3882, *P*_period_ = 0.2387 (1st day); *F*_period_ = 1.0805, *P*_period_ = 0.3679 (2nd day); *F*_period_ = 0.2966, *P*_period_ = 0.5918 (cumulative total)] did not affect the number of VPs emitted by cotton plants.

There was an interaction between the factors: aphid density *versus* cotton variety *versus* light/dark phase (*F* = 7.7295, *P* = 0.04150) for the number of VPs observed during the 3rd day of exposure of Bt cotton plants and non-Bt to aphids. It was found that on the third day, there was a higher VP production by non-Bt cotton plants exposed to 30 aphids/plant density than Bt cotton plants exposed to the same density during the photophase (Table 2). In addition, VP production by Bt and non-Bt cotton plants exposed to 60 and 30 aphids/plant, respectively, was higher in the light phase than in the dark phase (Table 2).

**Table 2:** Number of variation potentials emitted (mean ± SE) by Bt and non-Bt cotton plants exposed to different aphid densities / cotton plant and photophase and scotophase on the 3rd assessment day.

Variety	Density (aphid/plant)	Photophase	Scotophase
non Bt Cotton	30	6.75 ± 1.43 A a	2.50 ± 1.50 A b
Bt cotton		1.50 ± 0.86 B a	1.75 ± 1.03 A a
non Bt Cotton	60	3.25 ± 1.18 AB a	4.50 ± 1.18 A a
Bt cotton		5.00 ± 0.70 AB a	2.00 ± 1.08 A b
non Bt Cotton	0 (control)	0.25 ± 0.25 ^*nic*^	0.25 ± 0.25 ^*nic*^
Bt cotton		0.00 ± 0.00 ^*nic*^	0.25 ± 0.25 ^*nic*^

Uppercase letters compare averages within each column. and lowercase letters compare averages within each row. Means followed by the same letters do not differ from each other by overlapping confidence intervals generated by the quasi-Poisson model (*P* <0.05). ^*nia*^ = not incorporated in the analysis, because the absence of variability.

### Dispersal pattern of *A*. *gossypii* on Bt and non-Bt cotton plants

**Fig 5:**
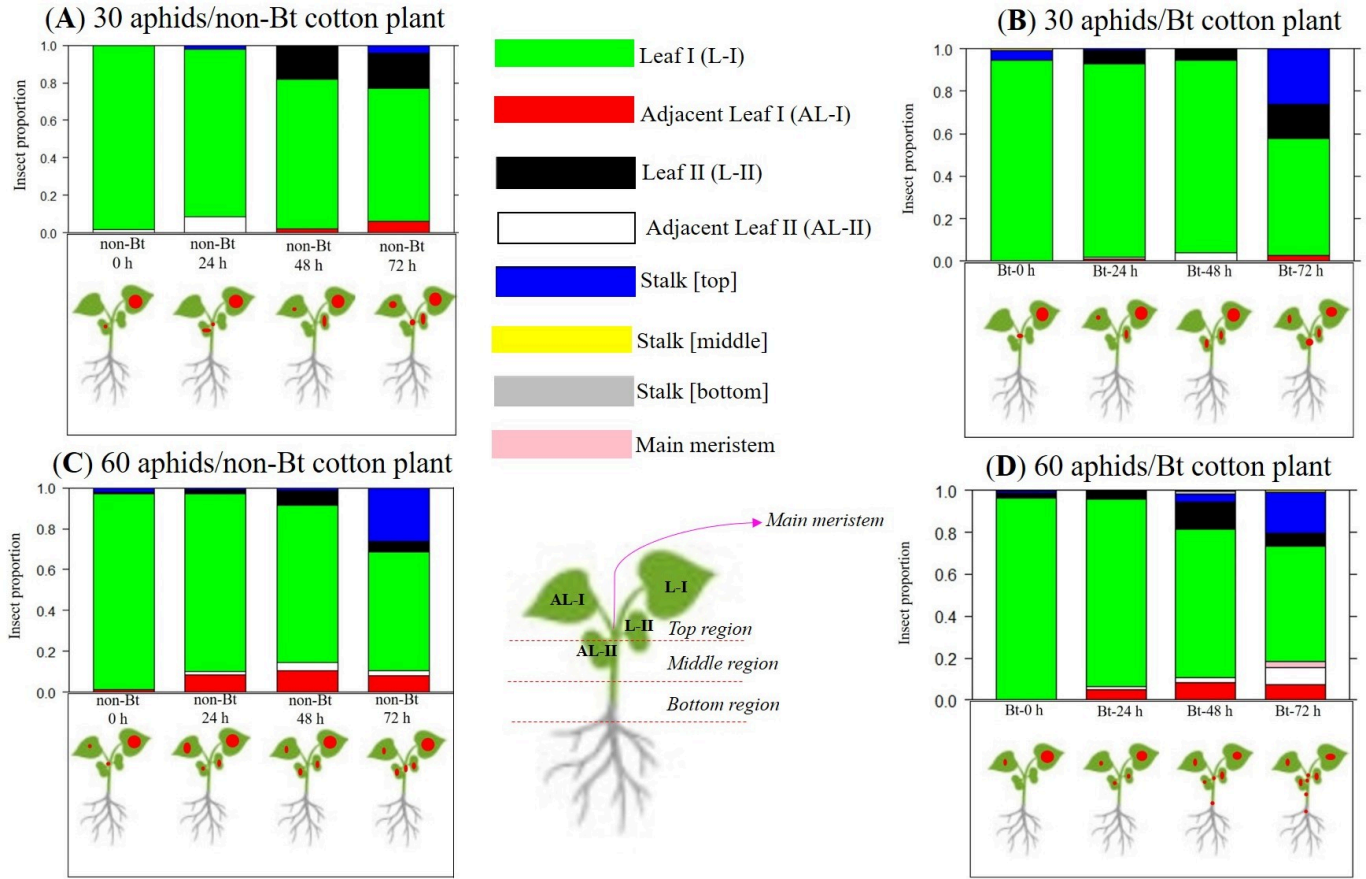
Multinomial distribution with occurrence rate of *A*. *gossypii* in the regions of non-Bt and Bt cotton plants at infestation times of 0 h, 24, 48 and 72 h with densities of 30 and 60 aphid/cotton plants. The red circle diameter represents the intensity of aphid infestation on cotton plants.

The behaviour of *A*. *gossypii*, independent of the factors: exposure time of plants to aphids, aphid density or cotton variety, followed a highly within-plant aggregated distribution pattern (*k* < 2) (Table 3, Fig 5A–5D).

**Table 3:** Confidence intervals associated with the *A*. *gossypii* aggregation index (95% CI) in Bt and non-Bt cotton plants submitted to densities of 30 and 60 aphids/cotton plants quantified at 0. 24. 48 and 72 h after the infestation of cotton plants with aphids.

Density/Variety	Time (h)			
	0	24	48	72
30 aphids/non-Bt cotton/	0.42–0.52 A b	0.47–0.74 A ab	0.55–0.85 A a	0.58–0.77 B a
60 aphids/non-Bt cotton	0.45–0.73 A b	0.55–1.67 A ab	0.78–3.03 A a	0.67–1.48 AB ab
30 aphids/Bt cotton	0.45–1.16 A a	0.45–0.83 A a	0.57–0.87 A a	0.77–1.55 AB a
60 aphids/Bt cotton	0.42–0.90 A b	0.63–0.80 A b	0.70–1.05 A b	1.12–1.46 A a

Uppercase letters compare averages within each column. and lowercase letters compare averages within each row. Means followed by the same letters do not differ from each other by overlapping confidence intervals generated by Boostrap (*P* <0.05).

Comparisons of the *k* index, based on confidence interval values, revealed that the highest *k* index of aphid aggregation with 30 aphids/non-Bt cotton plants was found at 48 h and 72 h after infestation of cotton plants with *A*. *gossypii* (Table 3). However, non-Bt cotton plants exposed to that density had a lower aphid *k* aggregation index at 72 h of infestation in relation to Bt cotton with 60 aphids/plant (Table 3).

The dispersal rate of *A*. *gossypii* was higher on Bt cotton with 60 aphids/plant than on non-Bt cotton plants with 30 aphids/plant at 72 h (Table 3). In fact, according to the multinomial distribution in the within-plant distribution of *A*. *gossypii*, it was confirmed that with 30 aphids/non-Bt cotton plants at 72 h, the highest proportions of aphids were on the adaxial (0.18) and abaxial (0.49) regions of the leaf (leaf I); however, there was increased insect dispersal to other positions, such as leaf II, adjacent leaf I and main meristem (Fig 5A). On the other hand, on Bt cotton plants infested with 60 aphids/plant, we observed the most dispersal pattern with 72 h of infestation, where there was clearly an increased insect dispersal, with 0.16 and 0.41 of aphids found in the adaxial and abaxial regions of leaf I, respectively, and 0.20 in the main meristem of the cotton plant.

No significant difference was observed among the treatments within the infestation times of 0 h, 24 h and 48 h in relation to the *k* index and multinomial distribution, except for the treatment with 60 aphids/Bt cotton plants within 48 h, which showed the most dispersal behaviour because it reached more regions of the cotton plants (Fig 5D).

In the comparisons of aggregation level among the time intervals within non-Bt cotton exposed to 60 aphids/plant, we perceived that the highest *k* aggregation index was during the infestation time of 48 h (Table 3). With Bt cotton plants at a density of 30 aphids/cotton plants, it was found that there was no significant alterations in the aphid dispersal pattern at all time intervals (Table 3).

## Discussion

The results from our study showed that both cotton varieties (Bt and non-Bt), when attacked by *A*. *gossypii*, emitted electrical signals of the variation potential type. Abiotic and biotic wounds are perceived differently by plants, as has been shown by other studies on plant-herbivore interactions [6, 21, 22]. Insect damage in plants plays a vital role to recognize the type of biotic stress to the plant [23, 24]. Plants differentiate herbivory from mechanical damage by recognizing compounds present in insect saliva because oral secretion of herbivores can induce ionic flux and promote depolarization of the plant membrane potential [25].

Here, in our research, it was possible to describe how Bt and non-Bt cotton plants react to *A*. *gossypii* stress by changing the transmembrane potential by recording extracellular electrical signals. Although plant responses to herbivorous attack are complex and involve a number of signals, it is important to note that different types of stimuli caused by insect action against plants trigger characteristic electrical signals evoked by plants with a specific influence on plant physiological processes [26]. The cascade of events involved in plant signalling as a function of stress perception begins at the plasma membrane of cells with changes in transmembrane potential or ion flow; these are the first responses of plants to biotic and abiotic stresses [27]. Attack on herbivorous plants is known to promote membrane potential changes that trigger an electrical signal that can travel to the entire plant or even trigger local plant defence mechanisms [28].

Our results indicate the presence of VP on Bt and non-Bt cotton plants at all assessed interval times. An aphid continuously inserts its buccal apparatus into the phloem vessels, altering the hydrostatic pressure in these vessels. VP is a signal whose propagation properties vary with the intensity and distance of the stimulus site and is probably a local electrical response, which is induced by a hydraulic signal, chemical signal or the combined action of these signals [29]. The hydraulic signal is a wave that results from increased hydraulic pressure in the plant, which propagates through the xylem and initiates the generation of a VP by triggering mechanosensitive ion channels present in the cells adjacent to the plant xylem vessels [30]. Therefore, harmful stimuli such as local damage, burning and mechanical injuries can evoke VPs [31, 32]. These kinds of electrical signals emitted by plants when under stress are especially important for hazard perception and response; thus, the plant can become able to mount an appropriate defence response [33].

Distinct response patterns were attributed to the perception and response to *A*. *gossypii* by each cotton variety and aphid density used in the research. Although we reported the first emission of signals on Bt cotton plants, there was a delay in terms of the propagated signal amount on Bt cotton plants with 60 aphids of infestation with *A*. *gossypii*, which produced the smallest numbers of signals between 0 to 36 h. Another important result was the greater dispersal behaviour related to this same treatment, mainly during and after 48 h of infestation. We suggest that the results could be supported by two hypotheses and explained independently or combined.

The first hypothesis is based on the possibility of a trade-off in terms of the defence of the Bt plant; a high dispersal could mean a larger exploitation of food resources by aphids and ease penetration of mouth apparatuses by aphids on Bt cotton plants, which may explain why Bt cotton plants emitted faster electrical signals than non-Bt cotton plants in the first moment, showing that Bt cotton plants may be more susceptible to aphid stress. Inducibility of a plant stress response is the ability to respond to stress only on demand. This is a strategy that is considered cost‐saving [34]. Therefore, this inducibility of plant defence may indicate a delay in the operation of defensive mechanisms; or on the other hand, may also mean a strategy to save energy and prevent self-poisoning [35], because it is possible that the Bt cotton plants may save energy with less production of signal until 36 h and producing them later. Since induction of a stress response implies that the plant starts activating resistance mechanisms upon encounter with the stressor, this strategy may lead to delay in mounting an effective response [36, 37], a first stress experience may prime the organism for an improved response to a subsequent stress.

With an electrical penetration graph (EPG) used for monitoring the penetration of the mouth apparatus by aphids on Bt and non-Bt plants and recording the waveforms that reflect different aphid feeding activities, a lower percentage of waveform np (non-penetration) was observed when the aphid was walking or grabbing the food with the rostrum on Bt cotton plants [38]. This suggests that aphids spend less time finding suitable places for penetration of their mouthparts on Bt cotton plants, probably due to the suitability of the tissue structure of Bt cotton plants to feed these aphids [38].

The second hypothesis is that the higher aphid dispersal on Bt cotton plants may indicate that the first signals emitted by the Bt cotton plants, even in smaller numbers than the non-Bt cotton plants, were enough to activate the Bt cotton plants’ defence, which prevented or hindered aphid feeding, such as occlusion of phloem sieve elements (SEs), which are the main conductive cells in the phloem, by clogging the sieved pores [39]. This is presumed to prevent sap loss [40, 41], and this process is seen as a primary plant defence response [39]. At the same time, the saliva constituents of sucking insects affect cellular processes [42] and therefore are perceived by cells, leading to the activation of signalling mechanisms, supporting the supposition that local damage induces the propagation of a specific injury substance through the xylem, and this induces the electrical response [43]. The main candidates for signalling molecules are H_2_O_2_ [44] systemin [45] jasmonic acid, abscisic acid, glutamate, among others. Both H_2_O_2_ and glutamate may activate calcium permeable channels, increasing intracellular calcium concentrations in plants [46] and being an important trigger for the generation and propagation of VP in plants [47].

The observed delay in the quantitative signalling pattern of Bt cotton plants when exposed to 60 aphids/plant could be attributed to self-preservation under stress and may be supported by the inclusion of resource reallocation for the production of metabolites and defensive structures (first and second hypotheses simultaneously). In the supplementary material, we show that almost twice the amount of Cry1F protein was observed on Bt cotton plants in the presence of aphids than in the absence of the insect, this result may be related with the fact that Bt cotton plants were faster to propagate VP signals. Well-known anti-herbivorous defence proteins include proteinase inhibitors (PIs) and polyphenol oxidases (PPOs), both of which are considered to interfere with digestive processes in herbivore intestines [35]. Therefore, simultaneous resource reallocation can serve not only to save resources; thus, the plant can subsequently use them for growth and reproduction but also to deprive the herbivore of food and consequently increase aphid dispersal, as observed in our results on Bt cotton with 60 aphids/plant, in order to search for the available food source. These above-mentioned hypotheses generate a basis for further studies that seek to highlight what possible defence mechanisms are involved and how effective they may be as a function of Bt and non-Bt cotton varieties.

In conclusion, the stress caused by the aphid *A*. *gossypii* was sufficient to trigger specific responses on Bt and non-Bt plants. Bt cotton plants were faster to propagate VP signals; however, they produced the signals in a smaller quantity with the highest aphid density, also promoting greatest within-plant aphid dispersal. Our results may guide future studies, which aim to elucidate the factors involved in the resistance to stress and plant defence processes and thus assist in the development of successful strategies in integrated pest management.

## Supporting information

S1 File (DOCX)

S1 Data (RAR)
