# Supplementary material for: Electrical signalling on Bt and non-Bt cotton plants under stress by Aphis gossypii
Source: PLoS One. 2021 Apr 8;16(4):e0249699. doi: 10.1371/journal.pone.0249699 (PMC8031172; doi:10.1371/journal.pone.0249699)
Supplement: S1 File — (DOCX) [file pone.0249699.s001.docx]

Electrical signalling on Bt and non-Bt cotton plants under stress by *Aphis gossypii*

Jéssica K. S Pachú^1*^, Francynes C.O. Macedo^2^, José B Malaquias^3^, Francisco S. Ramalho^4^, Ricardo F. Oliveira^2^, Flávia Pereira Franco^1^ and Wesley A. C. Godoy^1^

^1*^Corresponding author: Jéssica K. S Pachú, Rua Dr Alvim, 2227, Piracicaba, 13418-060, São Paulo, Brazil, Email: [jessikapachu@gmail.com](mailto:jessikapachu@gmail.com)

^1^Department of Entomology and Acarology, Luiz de Queiroz College of Agriculture (ESALQ), University of São Paulo (USP), Av. Pádua Dias 11, Piracicaba, 13418-900, São Paulo, Brazil,

^2^Department of Plant Physiology and Biochemistry, Luiz de Queiroz College of Agriculture (ESALQ), University of São Paulo (USP), Av. Pádua Dias 11, Piracicaba, 13418-900, São Paulo, Brazil.

^3^Department of Biostatistics, São Paulo State University (UNESP), Botucatu, Brazil.

^4^Biological Control Unit ⁄ Embrapa Algodão, Av. Osvaldo Cruz, 1143 Campina Grande, Paraíba 58107-720, Brazil.

**Determination of Cry1F protein content - Methods**

The Cry1F protein was quantified in Bt cotton plants in the presence of aphids (*A. gossypii* treatment) and in the absence of the insect (control). The plants were infested with the aphids with a paintbrush. We used the following 2 treatments: a1. Bt cotton plants infested with 60 aphids/plant, and a2. Bt cotton without aphid (Bt cotton control), distributed in 10 blocks = 10 plants/treatment.

The Cry1F protein concentrations in cotton leaf extracts were determined by immunological analysis (ELISA) using the Bt-Cry1F ELISA Kit (Quantitative DAS ELISA for the detection of the Bt-Cry1F transgenic protein, Catalog number: PSP 11700, Agdia^®^) according to the manufacturer’s instructions. The leaves tissue of each subsample was individually homogenized using a clean mortar and pestle and 2 ml of PBST (Phosphate Buffered Saline with Tween 20 Detergent) extraction buffer (137 mM NaCl, 2.7 mM KCl, 10 mM Na_2_HPO_4_, and 1.8 mM KH_2_PO_4_, 0.05% Tween® 20 detergent, pH 7.4). The supernatant was collected after centrifugation at 11,180×g at 4◦C for 20 min.

Enzyme conjugates (100 μl) were dispensed per well, and the same amount of each prepared sample, positive control, negative control and PBST buffer were dispensed in their respective wells. The standard Cry1F insecticidal protein, controls and samples were placed inside an humid box and incubated at 28°C for 60 minutes. After incubation the plate was washed using 1X PBST. After wash, 100 μl of the TMB substrate solution was added into each well and the plate was incubated in the dark for 20 min. The absorbance was recorded at 650 nm using Gen5 2.05 Software. Cry1Ac protein concentrations was calculated using the standard Cry1F insecticidal protein curve.

**Results**

Our results showed differences in Cry1F concentration on Bt cotton leaves of *A. gossypii*-infested and control (non-infested) plants (**Table I**).

**Table I** – Cry1F concentration (ng/ml) on non-infested (control) WideStrike cotton plants infested with *Aphis gossypii*

| Treatment | Cry1F concentration (ηg/ml) |
| --- | --- |
| Control (absence of aphids) | 4.38±0.67 b |
| *A. gossypii* | 7.85±0.28 a |
| *P_adjusted_* | = 0.0163 |

There was a difference between the treatments (*P* *<* 0.05).
